# The Potential Use of Volatile Biomarkers for Malaria Diagnosis

**DOI:** 10.3390/diagnostics11122244

**Published:** 2021-11-30

**Authors:** Hwa Chia Chai, Kek Heng Chua

**Affiliations:** Department of Biomedical Science, Faculty of Medicine, University of Malaya, Kuala Lumpur 50603, Malaysia; khchua@um.edu.my

**Keywords:** malaria diagnosis, *Plasmodium*, odor, volatile, non-invasive

## Abstract

Pathogens may change the odor and odor-related biting behavior of the vector and host to enhance pathogen transmission. In recent years, volatile biomarker investigations have emerged to identify odors that are differentially and specifically released by pathogens and plants, or the pathogen-infected or even cancer patients. Several studies have reported odors or volatile biomarkers specifically detected from the breath and skin of malaria-infected individuals. This review will discuss the potential use of these odors or volatile biomarkers for the diagnosis of malaria. This approach not only allows for the non-invasive mean of sample collection but also opens up the opportunity to develop a biosensor for malaria diagnosis in low-resource settings.

## 1. Introduction

Volatile organic compound (VOC)-based diagnostics have tremendous potential in becoming the next generation screening approaches for pathogen identification and infectious disease management. VOCs refer to low molecular weight metabolic compounds that easily evaporate at ambient temperatures due to their high vapor pressures and low boiling points. VOCs comprise a diverse group of carbon-based molecules, including alcohols, ketones, aldehydes, hydrocarbons, isocyanates, amines, terpenes and sulfides [1]. As a result of normal metabolic functions, a great number of VOCs are liberated from the healthy human body, produced via exhaled breath [2], skin secretions [3,4], saliva [5], blood [6,7], urine [2,8] and feces [9,10]. In the case of exhaled breath, a healthy human contains more than 1000 volatile organic compounds (VOCs), including isoprene, acetone, nitric oxide and methane [11,12]. Volatile samples collected from different parts of the body produce different compositions of VOCs. For example, the order in terms of the abundance of nitrogen-containing compounds is breath > skin secretions > urine > feces > saliva > milk > blood, while the order is skin secretions > breath > blood > feces > saliva > urine > milk for abundance of alcohols [11]. Many of these VOCs are probably generated by commensal microbes in the body and are often emitted and detectable through odor [13].

Increasing evidence shows that particular VOCs, or profiles of VOCs, may be unique to certain diseases or disease states. Pathogens can change both the quantity and composition of VOCs produced by patients during infections, and the VOCs detected in the headspace of infected cell cultures grown in vitro have proven that different pathogenic species may produce characteristic profiles of VOCs based on their distinct metabolisms [10,14,15,16]. Therefore, VOCs released by both the pathogens and infected hosts have potential value serving as a diagnostic signature for the identification of individuals with infection and discerning infection status. This in turn may provide an effective means of rapid, non-invasive pathogen identification which thus enables early diagnosis and treatment. Besides infectious diseases, volatile profiles specific to certain non-communicable diseases have also been identified, such as those from cancers [17,18], asthma, chronic obstructive pulmonary disease (COPD) [17] and chronic kidney disease (CKD) [19].

## 2. Recent Progress in Using Volatile Biomarkers as a Diagnostic Tool

Non-invasive diagnosis of disease via detection and measurement of volatile biomarkers has long been of great interest in healthcare applications. Any interference in the normal functions of an organ or body system, or distribution of normal flora due to infections or diseases will cause the production of disease biomarkers deep in the body that eventually circulate in the blood and are excreted through several pathways, such as urine, saliva, sweat, sputum, breath and skin. The excreted biomarkers that are volatile create inspiration and the prospect of developing non-invasive methods for disease diagnosis [20].

With the goal of increasing sensitivity and discrimination, the use of dogs for various infectious and non-infectious diseases like *Helicobacter pylori* [21], different cancer types [22,23], hypoglycemia in diabetes mellitus patients [24], epileptic seizures [25] and COVID-19 [26,27] has been explored. Varying detection sensitivities and specificities have been reported in the use of sniffer dogs for disease detection, with several studies successfully demonstrating very high sensitivity and specificity (>90%) [28,29]. According to systematic reviews that investigated the viability of human cancer detection by animals, breath was the best sample type for early cancer diagnosis [20,30]. However, the exact volatile compounds or composition of volatile profiles that the dogs react to is a question and has become the limitation of this method.

In the case of malaria, several studies did show the differential emission of odors or VOCs by malaria-infected individuals compared to those uninfected (Tables 1–3). Lindsay et al., attempted malaria diagnosis using two trained dogs, sniffing socks harboring foot odors of malaria-infected and -uninfected school children [31]. The two dogs managed to achieve approximately 70% and 90% of detection sensitivity and specificity, respectively, and both dogs reacted correctly in the same way to 93.9% and 77.3% of uninfected and infected samples, respectively. The sensitivity of both dogs improved to 81.8% (95% CI: 59.1–104.5) in detecting samples with a parasitemia of 200 parasites/µL or greater, which fulfilled the threshold requirement of WHO for malaria diagnostics.

The recent introduction of a new versatile diagnostic tool known as the electronic nose (E-nose) has shown significant promise in realizing the diagnosis of various diseases using VOCs for point-of-care settings [32]. It is a portable version of mass spectrometry that allows detection of VOCs’ composition on the breath or other volatile releasing samples. E-nose can be of various designs, but basically it refers to instruments consisting of an array of chemical sensors for detection of VOC profiles (smellprints) and an algorithm for pattern recognition [33]. A number of E-noses are already available on the market, such as Bloodhound BH-114 manufactured by Sensors Ltd. which has been used for bacterial infections [20,34], and JPL Enose developed by NASA, which has been used for brain cancer detection [20,35]. Upon training, E-nose is able to recognize individuals with the disease and locate the possible presence of the disease in specific tissues or compartments of the body based on the analysis of odor signatures containing specific mixtures of VOCs and other biomarker metabolites present in the samples. In a study by Capuano et al., which analyzed VOCs released by red blood cells (RBCs) infected by asexual and sexual stage cultures of *P. falciparum* relative to the uninfected RBCs, the results of proton transfer reaction time of flight mass spectrometry (PTR-ToF-MS) were substantially reproduced by a gas sensor array, which was claimed as an E-nose, despite the fact that E-nose was unable to differentiate between asexual stage *P. falciparum*-infected and uninfected RBCs [36].

## 3. Malaria-Associated VOCs

There are two possible routes in which malaria parasites can manipulate host odor: (i) direct signal emission from *Plasmodium* parasites or its interaction with RBCs, and (ii) indirect manipulation through alterations in skin microbial composition that eventually affect the host’s skin odor profile [37]. Freshly secreted human sweat is odorless [38] and only has a limited attraction to *Anopheles gambiae* compared to sweat incubated with skin bacteria [39,40]. Furthermore, a strong correlation is evidenced between human body odor and the species composition of skin bacteria [39,41,42,43,44]. Emanations from feet generally appear to be greatly attractive to *An. gambiae* compared to those from other parts of the body such as the hands and sweat [40]. The alterations of odor profiles of malaria-infected individuals due to emissions from *Plasmodium* parasites or changes in skin microbiota may serve as potential biomarkers for the development of the volatile-based diagnosis of malaria. 

### 3.1. Volatile Organic Compounds (VOCs) from Malaria Patients 

VOCs are normally identified and analyzed using gas chromatography-mass spectrometry (GC/MS). Limited studies can be found on describing the VOCs emitted from malaria patients. In a study involving children infected with malaria in Malawi, some breath volatiles were identified and a cumulative abundance of as few as six VOCs was able to differentiate between malaria-positive (*P. falciparum*) and -negative children, with a classification accuracy of 83%, specificity of 94% and sensitivity of 71% [45]. The six VOCs include methyl undecane, dimethyl decane, trimethyl hexane, nonanal, isoprene and tridecane (Table 1). In another study from Australia which also involved breath volatiles, nine compounds whose concentrations varied significantly over the course of malaria were identified: carbon dioxide, isoprene, acetone, benzene, cyclohexanone and four thioethers [46] (Table 1). The thioethers consisted of allyl methyl sulfide, 1-methylthio-propane, (Z)-1-methylthio-1-propene and (E)-1-methylthio-1-propene and were associated with parasitemia, given that their volatile levels declined accordingly after administration of the antimalarial drug. The four thioethers were also able to detect either submicroscopic or asymptomatic infections which have low parasitemia. However, the diurnal cyclical change of levels and significantly higher levels in only *P. falciparum*-infected individuals not only complicates the use of these thioesters as volatile biomarkers but also limits their use for detection of other *Plasmodium* species [46,47]. Hence, the same group of researchers looked for new volatile biomarkers and found that a set of terpenes, which do not have a diurnal change of levels, increased significantly with malaria infection. The accuracies of predicting *P. vivax* and *P. falciparum* using breath terpenes were up to 91% and 93.5%, respectively [47] (Table 1).

A study from Kenya revealed volatile changes on the skin (foot and arm) of malaria-infected individuals, as well as significant divergence in volatile profiles between symptomatic and asymptomatic infections [48]. They used machine learning algorithms to characterize the volatile signatures associated with each category of infection status and used them to develop predictive models for infection status classification. The compounds selected as predictors include toluene, hexanal, ethylcyclohexane, 4-hydroxy-4-methylpentan-2-one, ethylbenzene, propylcyclohexane, 2-ethylhexan-1-ol, nonanal and two unidentified compounds (Table 1). Models based on foot volatiles seemed to have higher sensitivity and accuracy compared to arm volatiles in discerning individuals with malaria regardless of infection status (sensitivity 95%; accuracy 77%); symptomatic infection (sensitivity 91%; accuracy 85%); asymptomatic infection (sensitivity 100%; accuracy 78%); and submicroscopic symptomatic and asymptomatic infections (both sensitivities 100%; both accuracies 100%). Thus, the authors claimed that their models can predict the infection status of human subjects with higher sensitivity than RDT and PCR, even in the case of low parasitemia [48]. Using the volatile data, the same group of researchers further identified the skin (arm or foot) VOCs specifically present in symptomatic schoolchildren who tested positive for malaria but not in malaria-negative children presenting similar (malaria-like) symptoms [49]. Predictive models consisting of various sets of VOCs managed to identify malaria-infected children presenting any symptoms such as fever and diarrhea with accuracies of 75%, 100% and 75%, respectively using arm VOCs, while accuracies using foot VOCs were 66.7%, 100% and 75%, respectively (Table 1).

**Table 1:** Malaria-associated VOCs found in infected individuals.

Study	Sample	VOCs	Sensitivity (SS)/ Specificity (SP)/ Accuracy (A)
Schaber et al., 2018 [45]	Breath	Methyl undecane Dimethyl decane Trimethyl hexane Nonanal ^§^ Isoprene * Tridecane	*P. falciparum*: 71% (SS), 94% (SP), 83% (A)
Berna et al., 2015 [46] Berna et al., 2018 [47]	Breath	Carbon dioxide Isoprene * Acetone Benzene Cyclohexanone4 thioesters: - Allyl methyl sulfide - Methylthio-propane (MTP) - Z-1-methylthio-1-propene (MTPNZ) - E-1-methylthio-1-propene (MTPNE)	
Berna et al., 2018 [47]	Breath	Terpenes: - Alpha-terpinene - M-cymene - Limonene - Terpinolene - 2 Unknown	*P. vivax*: Up to 91% (A) - Terpinolene: 91% (A) - M-cymene: 75.8% (A) *P. falciparum*: Up to 87.7% (A) - Terpinolene: 87.7% (A) - M-cymene: 92.7% (A)
De Moraes et al., 2018 [48]	Arm and foot volatiles	**Toluene** Octane **Hexanal**^**‡**^ 2,4-dimethylheptane **Ethyl cyclohexane** 2,4-dimethylhept-1-ene **4-hydroxy-4-methylpentan-2-one**^Φ^ **Ethylbenzene** *m*-xylene or *p*-xylene *o*-xylene **Propylcyclohexane** 1-ethyl-3-methylbenzene Benzaldehyde 1,2,4-trimethylbenzene Decane Octanal *S*(-)-limonene **2-ethylhexan-1-ol** **Nonanal**^§^ Dodecane **2 unidentified compounds** (Compounds in bold were consistently important key compounds in predicting models and/or showed distinct emission patterns)	*Plasmodium* spp.: Arm volatiles: - Infection: 80% (SS); 92% (A) - Symptomatic infection: 89% (SS; A) - Asymptomatic infection: 78% (SS); 75% (A) - Submicroscopic symptomatic: 88% (SS); 80% (A) - Asymptomatic infections: both 100% (SS; A) Foot volatiles: - Infection: 95% (SS); 77% (A) - Symptomatic infection: 91% (SS); 85% (A) - Asymptomatic infection: 100% (SS); 78% (A) - Submicroscopic symptomatic and asymptomatic infections: both 100% (SS; A)
Pulido et al., 2021 [49]	Arm and foot volatiles	Toluene **Octane** **Hexanal** ^**‡**^ 2,4-dimethylheptane Ethyl cyclohexane 2,4-dimethylhept-1-ene **4-hydroxy-4-methylpentan-2-one** ^Φ^ Ethylbenzene *m*-xylene or *p*-xylene ***o*****-xylene** Propylcyclohexane 1-ethyl-3-methylbenzene Benzaldehyde 1,2,4-trimethylbenzene **Decane** **Octanal** *S*(-)-limonene **2-ethylhexan-1-ol** Nonanal Dodecane 2 unidentified compounds (Compounds in bold were important predictors of malaria status for children with fever/diarrhea)	*Plasmodium* spp.: Arm volatiles: - Any symptoms: 85.7% (SS); 60% (SP); 75% (A) - Fever: 100% (SS; SP; A) - Diarrhoea: 100% (SS); 50% (SP); 75% (A) Foot volatiles: - Any symptoms: 57.1% (SS); 80% (SP); 66.7% (A) - Fever: 100% (SS); 50% (SP); 83.3% (A) - Diarrhoea: 50% (SS); 100% (SP); 75% (A)

VOCs—volatile organic compounds; ^§^^,^ *^,^ **^‡^**^, Φ^ Overlapped volatile compounds found in different studies. Note that only the consistently important compounds are taken into consideration.

### 3.2. Mosquito Attractant VOCs from Malaria Patients

VOCs released by *Plasmodium* parasites or malaria patients as mosquito attractants are relatively more extensively investigated and reported. *Plasmodium*-infected children are shown to draw in more mosquitoes than parasite-free children [50]. Malaria can change the odor of patients in order to attract vector mosquito *Anopheles* and enhance transmission of *Plasmodium* parasites. The study involving Malawian children infected with malaria also found significantly increased breath levels of mosquito-attractant terpenes, α-pinene and 3-carene [45] (Table 2). Malaria-infected children in Kenya produced higher levels of the aldehydes heptanal, octanal and nonanal compared to uninfected children and detected by mosquito *An. coluzzii* antennae [50] (Table 2). Levels of the three aldehydes were parasite-density-dependent, while the other two unsaturated aldehydes, (E)-2-octenal and (E)-2-decenal, were also found to be significantly increased in the parasite-positive individuals relative to the parasite-negative group. The *P. falciparum*-infected cohort in the Netherlands showed significant differential emission of 2-ethyl hexanoic acid, 2-methylbutanal, 3-methylbutanal, 3-hydroxy-2-butanone, 6-methyl-5-hepten-2-one, 1-dodecene, dodecanal, sesquiterpene and methyl dodecanoate either before, during or after the infection was induced, in which increased emissions of 2- and 3-methylbutanal and 3-hydroxy-2-butanone are known to be produced by skin bacteria, suggesting that changes in skin microflora are a factor [51] (Table 2). The three compounds, together with 6-methyl-5-hepten-2-one, may also take part in modulating *Anopheles* mosquitoes’ differential attractiveness to *P. falciparum*-infected humans.

*Plasmodium* gametocytes, the parasite’s transmissible stage, have been evidenced to influence the behavior of *Anopheles* mosquitoes. Gametocytes are able to double the attractiveness of gametocyte-infected patients to malaria vectors as compared to people who are parasite free, harbor asexual stages, or have gametocytes at submicroscopic densities, by changing the odor profile of these patients [52,53]. In the same study from Kenya, the presence of microscopic gametocytes was been linked to the emanation of ketone 2-octanone from infected individuals [50] (Table 2). A *P. falciparum* metabolite, (E)-4-hydroxy-3-methyl-but-2-enyl pyrophosphate (HMBPP), which was found to be both directly and indirectly manipulated by vector behavior, was hypothesized to be emitted from gametocyte-infected persons [53,54]. By amplifying the release of specific aldehydes and monoterpenes, HMBPP increased the feeding rate and indirectly promoted the attraction of *An. gambiae* sensu stricto to RBCs. A short distance attractiveness of the mosquito *An. darlingi* particularly in patients carrying *P. vivax* gametocytes was also observed [55].

**Table 2:** VOCs emitted by malaria-infected individuals that enhance mosquito attraction.

Study	Sample	VOCs as Mosquito Attractants
Shaber et al., 2018 [45]	Breath	Terpenes: α-pinene 3-carene
Robinson et al., 2018 [50]	Foot volatiles	Aldehydes: Heptanal Octanal Nonanal (E)-2-octenal (E)-2-decenal
De Boer et al., 2017 [51]	Skin volatiles	2-ethyl hexanoic acid 2-methylbutanal 3-methylbutanal 3-hydroxy-2-butanone 6-methyl-5-hepten-2-one 1-dodecene Dodecanal Sesquiterpene Methyl dodecanoate

VOCs—volatile organic compounds.

### 3.3. VOCs from Plasmodium Parasites

VOCs emitted by *Plasmodium* parasites to attract their vector mosquitoes have also been extensively studied and these VOCs could also serve as biomarkers for malaria detection. The presence of terpenes in the headspace gas of *P. falciparum*-infected RBCs was reported, whereby the dominant malaria parasite-specific terpenes were 4,5,9,10-dehydroisolongifolene and 8,9-dehydro-9-formyl cycloisolongifolene [56] (Table 3). Besides, limonene and α-pinene were also identified to substantially stimulate the odorant receptor of *An. gambiae*, suggesting that these plant-like volatile compounds produced by *P. falciparum* can modulate the attraction of vector mosquitoes to hosts. While no VOCs were identified exclusively to extracellular vesicles derived from *P. falciparum*-infected RBCs, 1,2,3-propanetriol diacetate (diacetin) was found to be commonly present on extracellular vesicles from infected cultures, despite the parasitemia of the cultures [57] (Table 3). In addition, the study also demonstrated a high association of hexanal with supernatant from the ultracentrifugation of the infected RBCs. Hexanal was also found at higher concentration in gametocyte-infected RBCs, particularly gametocytes at stages IV and V, with respect to uninfected and asexual stage-infected RBCs using PTR-ToF-MS analysis [36] (Table 3). The same study also reported 54 peaks which represent the gametocyte-specific VOCs, while asexual stage-specific VOCs consisted of only nine peaks.

**Table 3:** VOCs emitted by in vitro cultures of *Plasmodium* parasites.

Study	VOCs
Kelly et al., 2015 [56]	Terpenes: 4,5,9,10-dehydroisolongifolene 8,9-dehydro-9-formyl cycloisolongifolene Limonene α-pinene
Correa et al., 2017 [57]	1,2,3-propanetriol diacetate (diacetin) Hexanal
Capuano et al., 2019 [36]	Hexanal (Gametocytes) 54 peaks (Gametocytes) 9 peaks (Asexual stage)

VOCs—volatile organic compounds.

## 4. Challenges and Limitations in Volatile Biosensors

Volatile biomarkers detection may indeed offer an easy-to-use and sample-to-result point-of-care setting for malaria diagnosis. Nevertheless, before this volatile-based diagnostic method can be employed in real settings, several limitations that lead to the challenges of this method need to be addressed to ensure the VOC signatures or biomarkers and the detection device are robust enough to produce accurate diagnosis in varying environmental conditions.

The identification and detection of VOC signatures or profiles in all studies thus far are still in preliminary stages, which were restricted to certain geographical areas or populations only and conducted on a small scale. Given that genetics and environmental factors, such as weather and diet, may affect the body odor of an individual and cause high intra- and inter-individual variation [58,59,60,61], the malaria-specific VOCs mentioned above may not apply to other populations harboring different sets of genes and living in different geographical areas. This is also reflected in the different compositions of VOCs reported by each study, although some VOCs do overlap across multiple studies, such as nonanal and hexanal [45,48,49,50]. The accuracy, specificity and sensitivity of detection models also vary across all studies. Hence, besides standardizing the method for VOC collection, a universal set of VOC signatures or profiles may be required and tested in a wider geographical scale in order to warrant the diagnostic robustness and reliability of these VOCs in malaria.

Despite the findings of terpinolene and m-cymene with high accuracy in detecting *P. falciparum-* and *P. vivax*-infected individuals [47], as well as the identification of hexanal as a gametocyte-specific VOC [36], most studies reported the malaria-specific VOCs as a complex of several VOCs rather than a single VOC. Further complication arose when different VOC profiles were found for different infection status or *Plasmodium* stages, such as symptomatic and asymptomatic infections [48,49], and infections with the presence of gametocytes and asexual stage *Plasmodium* [36,53,55]. No studies to date have investigated how the other three human *Plasmodium*, i.e., *P. knowlesi*, *P. ovale* and *P. malariae*, alter the odor profiles of infected vectors and hosts, not to mention the VOC signatures for diagnosis. We can foresee the need for a very long effort to identify VOC signatures for discrimination of patients infected with the five *Plasmodium* spp., and the mixed infections of multiple species may even bring the difficulties up to a higher level.

Another concern about the malaria-specific VOCs mentioned above is that some of them could be cross-associated with plants, insects, pollutants or other diseases. For instance, hexanal, which was frequently seen in the composition of the malaria-specific VOCs mentioned above, is an alkyl aldehyde naturally produced by all plants and is frequently used in food flavorings to restore the “fresh green” odor of fruits and vegetables that has been lost during processing [62]. It is also commonly used in the cosmetic industry [63]. Carryover of hexanal from the natural environment, cooking or cosmetics may cause false-positive results in malaria diagnosis. In addition, hexanal and the other aldehydes including heptanal and nonanal are also the major VOC biomarkers for cancers, such as lung cancer and breast cancer [64,65,66]. The sharing of VOCs biomarkers with other diseases may complicate the detection of malaria. However, one thing to note is that different diseases or infections may prefer using volatiles collected from different types of samples or body parts, for example, skin volatiles appeared to be more prominent and useful for malaria diagnosis compared to breath volatiles which were otherwise more suitable for the detection of lung cancers or respiratory diseases. Hence, selection of the best sample type or body part for volatile collection and rigorous inclusion of VOCs biomarkers in the detection model is crucial for producing results with high accuracy, specificity and sensitivity.

To carry out malaria diagnosis in low-resource settings, the volatile detection device needs to be small, light and portable enough to be handheld and carried along. E-nose is probably the device that meets the requirements so far. Although E-nose can provide a quantitative response to a comprehensive VOC profile, individual VOCs are not recognized in this situation. It lacks the information about VOCs detected and discrimination among samples. However, it may be trained to recognize individual chemical compounds when in pure form or in simple gas sample compositions [67]. New-generation e-nose instruments have been improved to have both volatile-profiling capabilities as well as chemical analysis capabilities so that the composition of smellprints can be distinguished for identification of possible disease biomarkers [67]. However, the training of E-nose instruments and the interpretation of VOC patterns using various statistical analyses and software applications could be a technical impedance for some of the researchers to embrace this technology due to the complex and pragmatic mode of calibration of these instruments [68]. Another technical issue to be overcome is the lack of standardized methodology for VOCs collection as a significant discrepancy of results was observed when different sampling methods such as expiratory flow rate, breath hold and anatomic dead space were employed in the same group of subjects [69].

Some other limitations of E-noses include their insensitivity to odorant substances detectable by the human nose, that they are influenced by the presence of water vapor in sample analytes (especially breath samples) and can be inactivated (overloaded or poisoned) by certain highly polar compounds [32,67,68,70,71].

## 5. Conclusions

Given that the investigation of the feasibility of volatile biomarkers for malaria diagnosis is still at its premature stage compared to other diseases, extensive studies are required to address the challenges in the rigorous identification and validation of volatile biomarkers that specifically and accurately distinguish individuals with and without malaria, as well as those with different infection statuses. With the rapid development and improvement of the sensitivity of analytical instruments, we envision the use of volatile biomarkers for malaria diagnosis in the field, not only for early treatment but also for monitoring disease epidemiology.
